# Atypical primary meningioma in the nasal septum with malignant transformation and distant metastasis

**DOI:** 10.1186/1471-2407-12-275

**Published:** 2012-07-03

**Authors:** Byoung Joon Baek, Jae–Min Shin, Chi Kyou Lee, Ji Hye Lee, Koen Hyeong Lee

**Affiliations:** 1 Department of Otolaryngology-Head and Neck Surgery, Soonchunhyang University College of Medicine, Cheonan Hospital, 23-20 Bongmyung-Dong, Cheonan, 330-721, Chungcheongnam-do, South Korea; 2 Department of Pathology, Soonchunhyang University College of Medicine, Cheonan, South Korea; 3 Sangdang Otorhinolaryngologic clinic, Cheongju, South Korea

**Keywords:** Extracranial meningiomas, Ectopic meningiomas, Atypical meningiomas, Nasal septum, Nasal cavity, Metastasis

## Abstract

**Background:**

Primary extracranial meningiomas (PEMs) originating from the nasal septum are extremely rare, as are extracranial metastases of meningiomas.

**Case presentation:**

A 44-year-old male presented with a 2-month history of left-side nasal obstruction and frequent episodes of epistaxis. A friable mass originating from the nasal septum was resected completely via an endoscopic endonasal approach. According to WHO criteria, the tumor was diagnosed as an atypical meningioma radiologically and histopathologically. Two years later, a tumor recurred at the primary site with the same histopathological findings, and the patient was given local external radiotherapy (6840 cGy in 38 fractions). Two months after this local recurrence, a left anterior chest wall mass and a left parietal area scalp mass were observed. The subcutaneous mass was resected and showed histological evidence of malignant transformation. Several months after the last operation, the patient died.

**Conclusions:**

We describe the clinical, radiological, and bio-pathological features of this unique case and review the literature on atypical PEMs originating in the nasal septum. To our knowledge, this is the first reported case of an atypical PEM originating from the nasal septum that recurred with malignant transformation and extracranial metastasis.

## Background

Meningiomas are relatively common entities, accounting for 13–18% of intracranial neoplasms in adults, but they are rarely seen in extracranial locations, such as the spine, skull convexities, neck, chest, shoulder, and peritoneum [1,2]. Meningiomas in these locations are called extracranial meningiomas, and they account for fewer than 2% of all meningiomas [3]. Extracranial meningiomas include both primary and secondary types, based on the absence or presence of intracranial attachments, respectively. Primary extracranial meningiomas (PEMs, ectopic meningiomas) arise ectopically within a given tissue with no evidence of direct attachment to the brain tissue. Of these PEMs, only 11.5% are encountered in the nasal cavity and paranasal sinuses [4], and one PEM originating from the nasal septum has been reported [5].

Atypical meningioma is a rare variant that comprises 5-15% of all meningiomas. The reported rate of malignant change in meningiomas is less than 7% [6]. Although metastases may be more frequent in PEMs than in intracranial meningiomas [7-9], metastatic meningiomas are extremely rare (0.1%) [10]. To our knowledge, this is the first reported case of an atypical PEM originating from the nasal septum with distant metastasis and ultimately fatal progression.

## Case presentation

**Figure 1:**
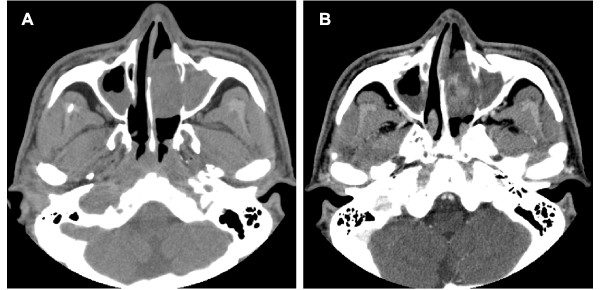
**Computed tomographic images before surgery.** (A) Non-contrast-enhanced PNS CT scan showing the primary lesion in the left nasal cavity. (B) The lesion showed heterogeneous enhancement on contrast-enhanced CT images.

**Figure 2:**
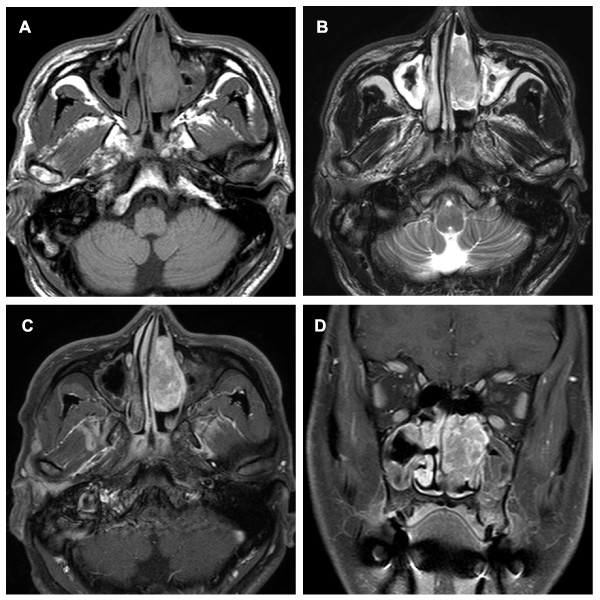
**Magnetic resonance images before surgery.** (A) T1-weighted PNS MRI showed a primary lesion of slightly lower signal intensity in the left nasal cavity. (B) The lesion was heterogeneously isointense on T2-weighted images. (C) Contrast-enhanced MRI showed heterogeneous enhancement of the lesion. (D) Coronal reconstruction of a T1-weighted MRI scan revealed an isolated primary lesion with no evidence of direct attachment to brain tissue.

A 44-year-old male was admitted to our clinic with a history of left-side nasal obstruction, episodes of epistaxis, hyposmia, and postnasal discharge over the previous 2 months. The patient had undergone endoscopic sinus surgery 2 years earlier for bilateral sinusitis at another clinic. Otherwise, his medical history was unremarkable. General and neurological examinations and routine laboratory test results were unremarkable. Non-contrast-enhanced computed tomography (CT) sections revealed an extensive hypodense non-calcified lesion occupying the left nasal cavity and pushing against the lateral nasal wall (Figure 1A). The lesion showed heterogeneous iodinated contrast enhancement and demonstrated remodeling of the adjacent bony structure (Figure 1B). Magnetic resonance imaging (MRI) showed that the tumor mass was of slightly lower intensity than the brain parenchyma on T1-weighted images and heterogeneously isointense on T2-weighted images, with significant heterogeneous contrast enhancement on gadolinium administration (Figure 2A-D). The preoperative CT and MRI showed an endonasal soft tissue mass with no intracranial connection. During the operation, a friable mass originating from the bony-cartilage junction of the nasal septum and extending to the lateral nasal wall was resected completely with the attached septal cartilage and nasal mucosa via an endoscopic endonasal approach. The safety margins were free of disease.

**Figure 3:**
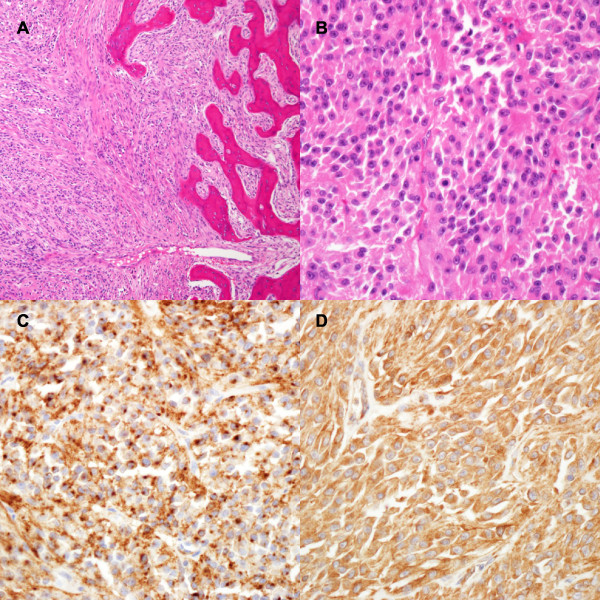
**Histopathology of the primary tumour.** (A) Uniform cells with a sheet-like or vague parallel fascicle-like growth involving the nasal septum (×100, H&E). (B) Atypical cells having round-to-oval nuclei, with prominent nucleoli and occasional mitoses (×400, H&E). The tumour cells were diffusely positive for EMA (C, ×400) and vimentin (D, ×400).

Microscopically, the tumor cells were arranged in sheet-like growths or were uninterrupted and patternless and showed nasal septum invasion (Figure 3A). No cellular whorl, psammoma body, or necrosis was observed. Individual tumor cells had round-to-oval nuclei, with prominent nucleoli and dense nuclear membranes. Mitosis was seen frequently (7/10 HFP, Figure 3B). The immunohistochemical profile was positive for epithelial membrane antigen (Figure 3C) and vimentin (Figure 3D), focal positive for S-100 protein, and negative for cytokeratin, p63, and smooth muscle actin. This histopathological investigation led to a diagnosis of an atypical meningioma (WHO Grade II) according to WHO criteria [11,12].

**Figure 4:**
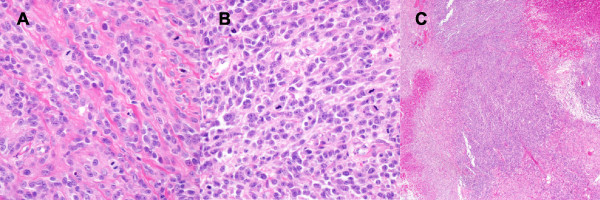
**Histopathology of the recurred and metastatic tumours.** (A) Atypical cells having round-to-oval nuclei, with prominent nucleoli and frequent mitoses in the nasal cavity recurring tumour. (B) The metastatic tumour had a higher mitotic index (up to 40 mitoses within 10 HPF) and Ki-67 labeling index (40%) than the primary tumour (×400, H&E). (C) Geographic tumoural necrosis and hemorrhage (×400, H&E).

Two years later, the patient was readmitted to the Hematology and Oncology Department of our hospital with recurrent severe epistaxis and general weakness, which had developed over the previous month. He also described breathing difficulties. Paranasal sinus CT showed recurrence of the left nasal cavity tumor, with palate and maxilla region involvement. Histological investigation of this second tumor showed morphological characteristics similar to the primary tumor (Figure 4A). Positron emission tomography/computed tomography (PET/CT) was used to identify distant metastases. PET/CT showed no abnormal glucose metabolic activity, except in the nasal cavity and palate region. The patient received local external radiotherapy (6840 cGy in 38 fractions) for local recurrence of meningioma.

Two months after local recurrence, subcutaneous tumors were found located on the left anterior chest wall (1.5 × 1.2 cm) and left parietal region (0.5 × 0.4 cm). The tumors were removed under local anesthesia. Histological examination showed morphological characteristics similar to the primary tumor (Figure 4B); however, the mitotic index (up to 40 mitoses within 10 HPF) and Ki-67 labeling index (40%) were higher than those of the primary counterpart. Foci of geographic necrosis were also found (Figure 4C). The tumor was classified as a malignant (WHO Grade III) meningioma.

After radiation therapy, the nasal cavity tumor shrank markedly. However, 2 weeks after the radiation therapy, multiple small subcutaneous tumors occurred over the entire body. At that time, the patient was very debilitated and did not want any other intensive treatment. He was transferred to a palliative care facility and died 3 months later.

## Conclusions

Primary extracranial meningiomas of the nasal cavity are extremely rare. To date, about 36 cases have been reported [5,11,12]. The origin of PEMs is unclear. PEMs are thought to arise from undifferentiated mesenchymal stem cells that get trapped or misplaced in intraosseous locations [13]. Conceivably, PEMs could arise from undifferentiated arachnoid cap cells associated with blood vessels or cranial nerves traversing the skull [14].

Atypical meningioma is a rare variant. According to WHO criteria, meningiomas are classified into three grades. Most meningiomas are benign (WHO Grade I); 5–15% of meningiomas are atypical (WHO Grade II); and 1–2% are anaplastic/malignant (WHO Grade III) [15].

Atypical meningiomas are diagnosed when increased mitotic activity (defined as ≥4 mitoses/10 HPF) or three or more of the following features are encountered: increased cellularity, small cells with a high nucleus:cytoplasm ratio, prominent nucleoli, uninterrupted patternless or sheet-like growth, and necrosis [16].

Malignant meningiomas (WHO Grade III) exhibit histological features of malignancy, including obviously malignant cytology (e.g., an appearance similar to sarcoma, carcinoma, or melanoma) and/or a high mitotic index (≥20 mitoses/10 HPF) [16]. Moreover, malignant meningiomas are usually fatal and have a higher rate of recurrence and metastasis [15] than the benign variant. Perry et al. [17] showed that the median survival time for malignant meningiomas was 1.5 years, with a 5-year mortality rate of 68%.

The incidence of metastases in meningiomas is very low. Although surgical removal may increase the risk of iatrogenic metastases of histologically aggressive meningiomas [18], malignant meningiomas can disseminate with no previous surgery [19]. The reported incidence of distant metastases in malignant meningioma is around 43% [20]. The mode of metastatic spread is unclear. Three possible dissemination patterns have been described: hematogenous, lymphatic, and via the CSF [20]. In PEMs, the likely routes of distant metastases are venous and lymphatic, because PEMs have no intracranial attachments. Metastasis via the venous system usually causes pulmonary, hepatic, and skeletal metastases, while that via the lymphatic system causes lymph node and subcutaneous metastases [21]. In this case, distant metastases were seen in the anterior chest wall and scalp 2 months after local recurrence. However, with time, multiple subcutaneous tumors developed over the entire body, so it is possible that distant metastasis to vital organs, such as the lungs or liver, occurred.

We presented the case of a middle-aged male with distant metastasis from a PEM of atypical histology originating from the nasal septum. Although classified histologically as atypical, the meningioma exhibited unusually aggressive behavior and transformed to a malignant meningioma after surgery. Additionally, it is known that meningiomas tend to become more histologically aggressive with each recurrence [21]. In our case, this clinical characteristic was seen.

Currently, complete surgical excision of PEMs is the treatment of choice and there is no need for adjuvant treatment [11]. However, our case showed that tumors invade adjacent septal cartilage, and it has been reported that adjuvant radiotherapy contributed significantly to improvements in overall survival and recurrence-free survival in intracranial meningiomas with local tissue invasion [22]. Given their anatomical complexity and the fact that they are covered with mucosal epithelium, which has many vascular and lymphatic channels, PEMs in the nasal cavity have a higher risk of incomplete tumor resection. Therefore, routine postoperative adjuvant radiotherapy and careful postoperative imaging may be necessary to improve the outcome in patients with atypical PEMs in the nasal cavity.

## Consent

Written informed consent was obtained from the patient’s relatives for publication of this case report and accompanying images. A copy is available for review from the Editor-in-Chief of this journal.

## Competing interests

The authors declare that they have no competing interests.

## Authors’ contributions

BJB conceptualized the case report and wrote the manuscript as a major contributor. JMS and KHL were involved in drafting the manuscript and revising it for intellectual content. JHL carried out the pathologic analysis. CKL was involved in collection of data. All authors read and approved the final manuscript.

## Pre-publication history

The pre-publication history for this paper can be accessed here:

http://www.biomedcentral.com/1471-2407/12/275/prepub
